# Systemic MEK inhibition enhances the efficacy of 5-aminolevulinic acid-photodynamic therapy

**DOI:** 10.1038/s41416-019-0586-3

**Published:** 2019-09-25

**Authors:** Vipin Shankar Chelakkot, Jayoti Som, Ema Yoshioka, Chantel P. Rice, Suzette G. Rutihinda, Kensuke Hirasawa

**Affiliations:** Division of BioMedical Sciences, Faculty of Medicine, Memorial University of Newfoundland, St. John’s, NL A1B 3V6 Canada

**Keywords:** Targeted therapies, Targeted therapies

## Abstract

**Background:**

Protoporphyrin IX (PpIX) gets accumulated preferentially in 5-aminolevulinic acid (5-ALA)-treated cancer cells. Photodynamic therapy (PDT) utilises the accumulated PpIX to trigger cell death by light-induced generation of reactive oxygen species (ROS). We previously demonstrated that oncogenic Ras/MEK decreases PpIX accumulation in cancer cells. Here, we investigated whether combined therapy with a MEK inhibitor would improve 5-ALA-PDT efficacy.

**Methods:**

Cancer cells and mice models of cancer were treated with 5-ALA-PDT, MEK inhibitor or both MEK inhibitor and 5-ALA-PDT, and treatment efficacies were evaluated.

**Results:**

Ras/MEK negatively regulates the cellular sensitivity to 5-ALA-PDT as cancer cells pre-treated with a MEK inhibitor were killed more efficiently by 5-ALA-PDT. MEK inhibition promoted 5-ALA-PDT-induced ROS generation and programmed cell death. Furthermore, the combination of 5-ALA-PDT and a systemic MEK inhibitor significantly suppressed tumour growth compared with either monotherapy in mouse models of cancer. Remarkably, 44% of mice bearing human colon tumours showed a complete response with the combined treatment.

**Conclusion:**

We demonstrate a novel strategy to promote 5-ALA-PDT efficacy by targeting a cell signalling pathway regulating its sensitivity. This preclinical study provides a strong basis for utilising MEK inhibitors, which are approved for treating cancers, to enhance 5-ALA-PDT efficacy in the clinic.

## Background

Photodynamic therapy (PDT) is a cancer treatment modality that utilises photosensitizers and light exposure to treat different types of cancers.^1,2^ Photosensitizers are selectively accumulated in cancer cells and are activated by exposure to light of specific wavelengths. This leads to the rapid generation of singlet oxygen and reactive oxygen species (ROS), resulting in cellular oxidation and programmed cell death (PCD).^3–5^ 5-Aminolevulinic acid (5-ALA) is a naturally occurring photosensitizer precursor, which is metabolically converted to a photosensitizer, protoporphyrin IX (PpIX), by enzymes of the haem biosynthesis pathway.

PDT utilising 5-ALA (5-ALA-PDT) was introduced into the clinics in the early 1990s to treat skin cancer,^6,7^ and has since been approved for treating other types of cancers, including biliary tract, bladder, brain, breast, colon, digestive tract, oesophagus, head and neck, lung, pancreas, prostate and skin cancers.^2^ As light exposure activates PpIX locally, 5-ALA-PDT can provide a focal, non-invasive treatment with less adverse effects compared with radiotherapy or chemotherapy.^1,2,8^ In addition, 5-ALA-PDT triggers cell death through multiple mechanisms involving various intracellular targets and provides significant tumour selectivity.^9,10^ However, the long-term recurrence rate for 5-ALA-PDT is relatively high, which limits its clinical applications.^11^ Previous studies have reported 20% and 35–45% disease recurrence in patients with oral carcinoma and squamous and basal cell carcinoma, respectively.^12–14^ One of the major causes of this incomplete response is low or sub-optimal PpIX accumulation in tumours.^15^ PpIX accumulation is dependent on the cell type, degree of transformation and intracellular iron content, resulting in inconsistent levels of PpIX in tumours.^2,16–18^ Moreover, PpIX undergoes rapid photo-bleaching with irradiation, which destroys the photosensitizer (PS) and limits the achievable amount of ROS. Thus, the treatment response is highly dependent on the initial PpIX concentration in the tumour.^10,19^ Therefore, it is essential to develop strategies to promote PpIX accumulation in tumours to enhance the therapeutic efficacy of 5-ALA-PDT.

The Ras/mitogen-activated protein kinase (MEK) pathway is one of the oncogenic signalling pathways that regulate cell proliferation, growth and death.^20,21^ Constitutive activation of the Ras/MEK pathway induced by activating mutations in its signalling components is common in cancer cells.^20–24^ Earlier studies have shown that oncogenic transformation increases 5-ALA-induced PpIX accumulation.^25,26^ Therefore, in our previous study, we investigated the mechanisms underlying Ras/MEK pathway-mediated regulation of PpIX accumulation in cancer cells.^27^ Unexpectedly, we observed that MEK lowered 5-ALA-induced PpIX accumulation in ~60–70% of human cancer cell lines.^27^ The increase in PpIX accumulation by MEK inhibition was cancer cell-specific, and was not observed in non-cancer cell lines. We also discovered that Ras/MEK activation reduced PpIX accumulation by increasing PpIX efflux through ATP-binding cassette transporter B1 (ABCB1), one of the PpIX efflux channels and ferrochelatase (FECH)-mediated PpIX conversion to haem. Most importantly, we demonstrated that treatment with MEK inhibitors could enhance PpIX fluorescence selectively in tumours, but not in healthy tissues in mouse models of cancer, suggesting that MEK inhibition facilitates the preferential enhancement of PpIX accumulation in tumours. These results indicate that the Ras/MEK pathway has opposing effects on PpIX accumulation in cancer cells, and its impact is more significant in reducing intracellular PpIX. Thus, the Ras/MEK pathway plays an intricate role in the regulation of PpIX accumulation in cancer cells.

As critical effectors in the Ras/MEK pathway, MEKs have become therapeutic targets for various cancers, including metastatic melanoma, pancreatic cancer, biliary tract cancer, non-small cell lung carcinoma (NSCLC), uveal melanoma and acute myeloid leukaemia.^28,29^ Two MEK inhibitors—trametinib and cobimetinib—have been approved for clinical use in BRAF-positive metastatic melanoma and NSCLC,^28^ and several other MEK inhibitors are currently in clinical development.^28^ Moreover, apart from monotherapy, chemotherapy and radiotherapy in combination with MEK inhibitors have shown promising results.^28,30,31^ Our previous study suggested that MEK inhibitors may also be useful in the context of 5-ALA-PDT; however, this is yet to be tested.

In this study, we tested the hypothesis that MEK inhibitors could be an effective partner for combined 5-ALA-PDT to achieve complete therapeutic responses. Specifically, we sought to determine the efficacy of 5-ALA-PDT combined with a MEK inhibitor in vitro and in vivo. The results from our study indicate that MEK inhibitors are promising candidates for clinical use in conjunction with 5-ALA-PDT, and should be further evaluated in preclinical and clinical trials.

## Methods

### Cells and reagents

Human glioma cell lines, U-118, U-251; colon cancer cell line DLD-1; lung cancer cell lines H-1299 and H460 and breast cancer cell lines Hs 578T and MDA-MB-251 were obtained from the American Type Culture Collection (Manassas, VA, USA). U-118 is a permanent cell line derived from a grade IV human glioblastoma—astrocytoma, and U-251 was derived from a grade III–IV human malignant glioblastoma multiforme. DLD-1 was derived from a Dukes’ type C, colorectal adenocarcinoma, H-1299 is an NSCLC cell line, H460 is a large cell lung cancer cell line, and Hs 578T and MDA-MB-231 are triple-negative breast cancer (TNBC) cell lines. All human cell lines used in the study were authenticated by STR DNA analysis (DDC Medical, Fairfield, OH, USA; Center for Applied Genomics, SickKids, Toronto, Canada). Mouse 4T1 mammary tumour cells were obtained from Dr Jean Marshall (Dalhousie University, Halifax, Canada). All cell lines were maintained in high glucose Dulbecco’s modified Eagle’s medium (DMEM) (Invitrogen, ON, Canada), supplemented with 10% foetal bovine serum (FBS) (Corning, VA, USA) and 1:100 Antibiotic–Antimycotic (×100; ThermoFisher Scientific). MEK inhibitors U0126 and selumetinib were purchased from Cell Signaling Technology (Danvers, MA, USA) and Selleckchem (Houston, TX, USA), respectively; and 5-Aminolevulinic acid from Sigma (Oakville, ON, Canada).

### PpIX measurement

Cells (5 × 10^4^/well) plated in 24-well plates were treated with U0126 or DMSO (control vehicle) for 20 h, and then with 5-ALA for 4 h. The cells were lysed using radioimmunoprecipitation assay (RIPA) buffer, and PpIX fluorescence in cell lysates was measured using a Synergy Mx Fluorescence plate reader (BioTek Instruments Inc. VT) with a 405 nm excitation/630- nm emission filter.

### In vitro 5-ALA-PDT

Cells (5000/well) plated in 96-well plates were treated with U0126 or DMSO for 20 h, and then with 5-ALA for 4 h. The cells were irradiated using a Theralase TLC 3000A modular light source (Theralase Technologies Inc., Toronto, Canada; *λ* = 618–630 nm, fluence rate = 150 mW/cm^2^, energy density (ED) = 27 J/cm^2^). Cell viability was measured 24 h after 5-ALA-PDT using the Colorimetric Cell Viability Kit I (WST-8) (PromoCell GmbH, Germany), following the manufacturer’s instructions. The pharmacological interactions between U0126 and 5-ALA-PDT were analysed using the Chou and Talalay method.^32–34^ Briefly, the proportion of cells dead (fraction affected, fa) or viable (fraction unaffected, fu = 1 − fa) at concentration D for each drug alone or together was determined experimentally, and the combination indices (CI) were calculated using the equation,

$${\mathrm{CI}} = \frac{{\left({\mathrm{ D}} \right)_1}}{{\left( {{\mathrm{Dx}}} \right)_1}} + \frac{{\left({\mathrm{ D}} \right)_2}}{{\left( {{\mathrm{Dx}}} \right)_2}}$$

where (D)_1_ and (D)_2_ are the concentrations of 5-ALA and U0126, respectively, that together induce x% reduction in cell viability. Dx is the concentration required for each drug alone that induces x% inhibition which was calculated using the equation,

$${{{\mathrm{Dx}}}} = {{{\mathrm{Dm}}}}\left[ {{{{\mathrm{fa}}}}/\left( {1 - {{{\mathrm{fa}}}}} \right)} \right]^{1/m},$$

where m is the slope and Dm is the x-intercept of log[fa/(1 − fa)] plotted against log (D). The CI is a quantitative definition of synergism (CI < 1), additive effect (CI = 1), or antagonism (CI > 1) in multidrug interactions.

### Biochemical analyses of 5-ALA-PDT-induced cell death

For evaluating changes in cell cycle progression and PCD-induced DNA fragmentation after PDT, the cells were harvested 4 h after 5-ALA-PDT, fixed and permeabilized with 70% cold ethanol, and then stained with propidium iodide (PI) solution (50 µg/ml PI in PBS with 550 U/ml RNaseA; Abcam, USA). Cellular DNA content was analysed by flow cytometry using a BD FACSCalibur (BD Biosciences, San Jose, CA, USA).^35^ The data were analysed using FlowJo (FlowJo LLC, OR). Western blot analysis was conducted to detect PCD markers (cleaved PARP, pro-caspase 3 and cleaved caspase 3) using the apoptosis western blot cocktail (ab136812, Abcam).^36^ The amount of cellular ROS was measured using the OxiSelect^TM^ Intracellular ROS assay kit (Cell Biolabs Inc. San Diego, CA, USA) following the manufacturer’s instructions. Briefly, 5-ALA-PDT-treated cells were incubated with 2′,7′-dichlorodihydrofluorescein diacetate (DCFH-DA) for 30 min at 37 °C, and the fluorescence was measured using a Synergy Mx Fluorescence plate reader (BioTek Instruments Inc., VT) at 480 nm/530 nm. The amount of ROS was determined by comparison with a 2′,7′-dichlorodihydrofluorescein (DCF) standard curve.

### Electron microscopy and ultrastructure studies

The cells were fixed in Karnovsky fixative (2% paraformaldehyde (Canemco-Marivac Inc. PQ, Canada) and 2.5% Glutaraldehyde (Canemco-Marivac Inc.) in 0.1 M sodium cacodylate buffer (Canemco-Marivac Inc.)) for 20 min, and post-fixed in 1% osmium tetroxide (Sigma-Aldrich, St. Louis, MO, USA) in 0.1 M sodium cacodylate (Canemco-Marivac Inc.) buffer pH 7.4. The fixed cells were dehydrated in increasing concentrations (70–100%) of ethanol and in acetone, and then embedded in BEEM resin capsules (Electron Microscopy Sciences, Hatfield, PA, USA). Ultrathin sections were mounted on 300 mesh copper grids, stained with uranyl acetate (Canemco-Marivac Inc.) and lead citrate (Canemco-Marivac Inc.), and observed using an FEI Tecnai G2 Spirit transmission electron microscope (FEI, Hillsboro, OR), operating at 80 KV.^37^

### In vivo 5-ALA-PDT

Female BALB/c mice and male athymic nude mice purchased from Charles River Laboratories (Montreal, QC, Canada) were housed in a barrier unit within the Central Animal Care Facility of the Health Sciences Center at Memorial University of Newfoundland. Animal experiment protocols were approved by the Institutional Animal Care Committee, and were in accordance with the guidelines of the Canadian Council on Animal Care. As shown in Supplementary Fig. S1, at 8 weeks of age, BALB/c female mice or athymic nude mice were injected subcutaneously into the right hind flanks with mouse mammary carcinoma 4T1 cells or human colon cancer DLD-1 cells suspended in PBS (2 × 10^6^ cells/100 µL), respectively. After the development of palpable tumours (3–5 mm in diameter), the mice were randomly assigned to one of the following four groups: (1) control, (2) selumetinib, (3) 5-ALA -PDT and (4) selumetinib/5-ALA-PDT (*n* = 15 per group for the 4T1 model and *n* = 9 per group for the DLD-1 model). The mice were orally (p.o.) administered a control vehicle (0.5% propyl methylcellulose in PBS) or selumetinib (150 mg/kg body weight (BW)). Six hours later, Groups 3 and 4 received 5-ALA (200 mg/kg BW) by intraperitoneal (i.p.) injections and were housed in the dark. Two hours after i.p. ALA, the mice were irradiated (ED = 40 J/cm^2^) using a LuxX 633-100 laser (Omicron Laserage, Germany) coupled with a frontal diffusor, FD1 (Medlight S.A, Switzerland). To ensure precise irradiation, the mice were anaesthetised by isoflurane inhalation and positioned horizontally on a heat mat, and the fibre was fixed vertically above the tumour. After irradiation, the mice were housed in the dark for 24 h. The tumours were measured every day to monitor tumour progression up to 60 days or until the endpoint (tumour measuring 15 mm on any one axis) was reached. The mice were sacrificed by carbon dioxide inhalation on reaching the endpoint. For histological studies, three mice from each group were sacrificed by carbon dioxide inhalation at 12 and 24 h after 5-ALA-PDT, and the tumours were resected.

### Histological evaluation

Formalin-fixed paraffin-embedded tumour sections were stained with haematoxylin and eosin (H&E) to observe morphological changes. PCD in the tumours was detected by TUNEL staining using the TUNEL Assay Kit (ab66110, Abcam), following the manufacturer’s instructions. The sections were counterstained with 4′,6-diamidino-2-phenylindole (DAPI) for observing the nuclei. Quantitative analysis of TUNEL-positive cells was performed on five randomly selected images from each mouse (three mice/group) using Fiji (NIH).

### Statistical analyses

Statistical analyses were performed using Prism 7.0 (GraphPad). One-way or two-way ANOVA was performed for multiple data sets. Median survival comparison was performed using the log-rank test. *p* < 0.01 and *p* < 0.05 were considered statistically significant for in vitro and in vivo experiments, respectively.

## Results

### Different human cancer cell lines have distinct 5-ALA-PDT-sensitivities

**Fig. 1:**
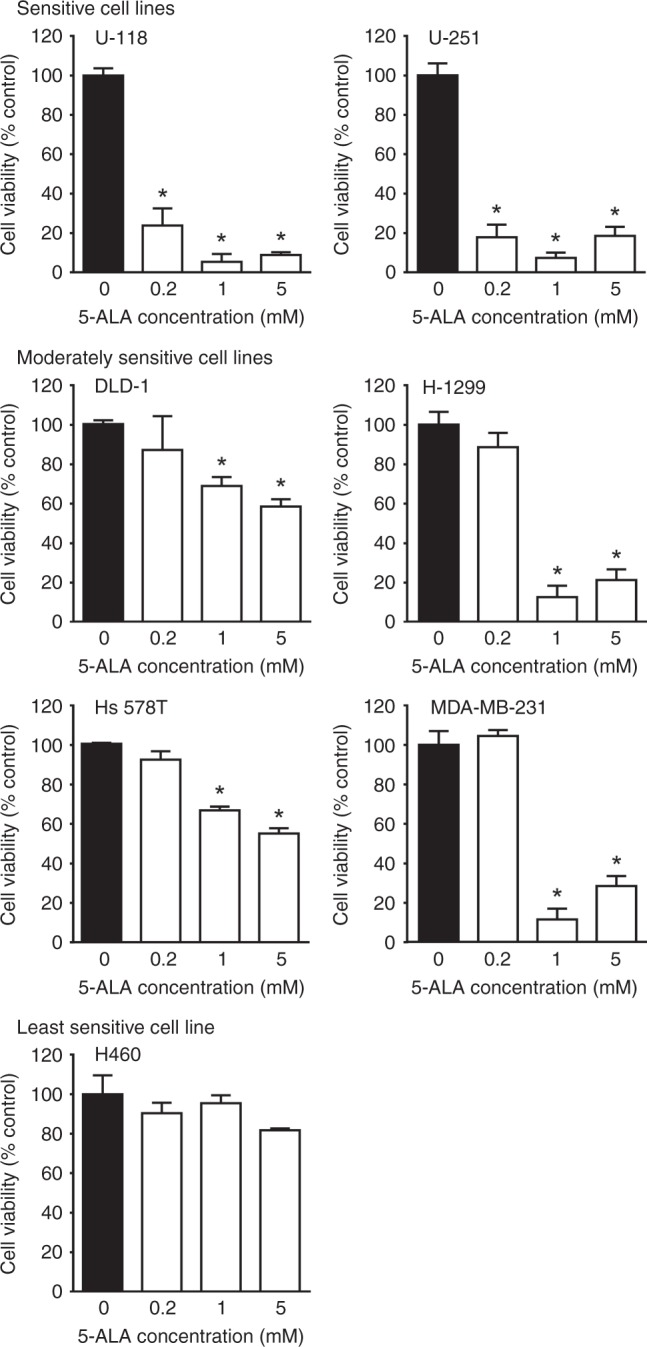
Sensitivity of human cancer cell lines to 5-ALA-PDT. Human cancer cells were treated with or without 5-ALA for 4 h and then irradiated. Mean ± SD of % cell viability at 24 h after PDT relative to controls (no 5-ALA, black bars) from three independent experiments is presented. *p < 0.01 vs. 0 mM 5-ALA by one-way ANOVA with Turkey’s post hoc test. Cell lines that were significantly killed by 5-ALA-PDT were classified as Sensitive cell lines. Cell lines that were significantly killed by 5-ALA-PDT at the highest tested 5-ALA concentration (5 mM) but not at the lowest concentration (0.2 mM) were classified as Moderately sensitive cell lines. A cell line that was not sensitive to any 5-ALA concentrations tested was classified as Least sensitive line

We tested seven human cancer cell lines, including human breast cancer (Hs 578T and MDA-MB-231), colon cancer (DLD-1), glioblastoma (U-118 and U-251) and lung cancer (H-1299 and H460), for their sensitivities to 5-ALA-PDT (Fig. 1). The cell lines were treated with different concentrations of 5-ALA (0, 0.2, 1 and 5 mM) for 4 h and then irradiated with a red laser. Cell viability was measured 24 h after 5-ALA-PDT. No cell death was observed in non-irradiated cells, suggesting that 5-ALA did not have any toxicity even at higher concentrations. 5-ALA-PDT effectively killed human glioblastoma cell lines (U-118 and U-215) at all 5-ALA concentrations that were tested (0.2, 1 and 5 mM) and were classified as 5-ALA-PDT-sensitive cell lines. The viabilities of DLD-1, H-1299, Hs 578T and MDA-MB-231 were significantly reduced by 5-ALA-PDT when treated with higher concentrations of 5-ALA (1 and 5 mM), but not with the low concentration (0.2 mM), and were classified as moderately sensitive cell lines. Finally, H460 was not responsive to 5-ALA-PDT at any concentrations we tested. This was the only cell line, which was classified as the least sensitive cell line.

### MEK inhibition promotes 5-ALA-PDT efficacy in vitro

**Fig. 2:**
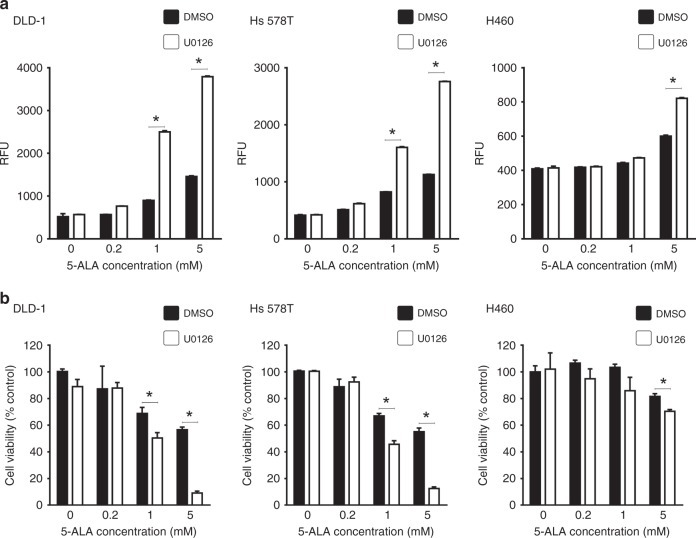
MEK inhibition increases 5-ALA-PDT sensitivity of moderately and least sensitive cell lines. **a** Cellular fluorescence in relative fluorescence unit (RFU) in moderately sensitive DLD-1 and Hs 578 T cells, and least sensitive H460 cells. **b** % Cell viability 24 h after PDT relative to controls (DMSO, no 5-ALA). The data presented as mean ± SD from three independent experiments. *p < 0.01 by two-way ANOVA with Turkey’s post hoc test

Previously, we demonstrated that MEK inhibition increases PpIX accumulation in different cancer cell lines treated with 5-ALA.^27^ As sufficient accumulation of PpIX is essential for effective cancer cell killing by 5-ALA-PDT,^3–5^ we sought to determine whether MEK inhibition would enhance 5-ALA-PDT efficacy. We used the MEK inhibitor U0126 to inhibit oncogenic Ras/MEK in the moderately sensitive (DLD-1 and Hs 578T) and the least sensitive (H460) cell lines. MEK inhibition significantly increased PpIX accumulation, as measured by its fluorescence, in all cell lines in a concentration-dependent manner (Fig. 2a). When cells pre-treated with U0126 were subjected to 5-ALA-PDT, we observed significantly increased cell death in the moderately sensitive cell lines (DLD-1 and Hs 578T) at 5-ALA concentrations of 1 and 5 mM (Fig. 2b). Furthermore, 5-ALA-PDT sensitivity of the least sensitive H460 cell line was also increased by U0126 pre-treatment (Fig. 2b). These results suggest that an active MEK pathway decreases 5-ALA-PDT efficacy in certain cancer cell lines.

**Fig. 3:**
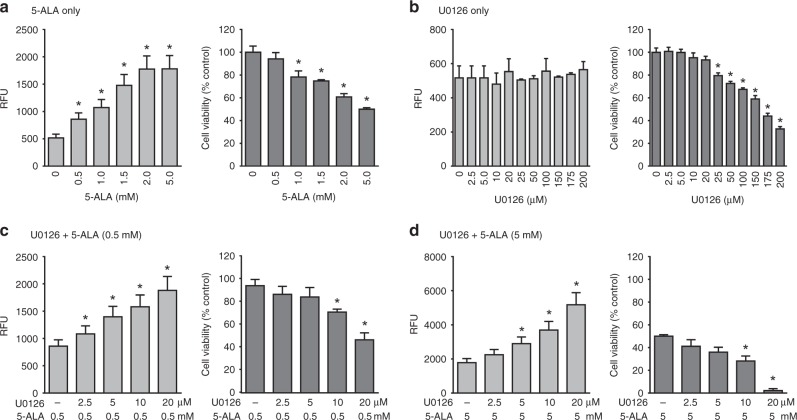
MEK inhibition promotes PpIX accumulation and 5-ALA-PDT efficacy in a concentration-dependent manner. Moderately sensitive DLD-1 cells were treated with (**a**) 5-ALA only, (**b**) U0126 only or (**c** and **d**) U0126 followed by 5-ALA. Mean ± SD of cellular fluorescence (left panels) and % cell viability relative to untreated cells (right panels) from three independent experiments are shown. *p < 0.01 by one-way ANOVA with Turkey’s post hoc test

To further characterise the effect of MEK inhibition on the efficacy of 5-ALA-PDT, we examined 5-ALA-PDT sensitivity of the moderately sensitive DLD-1 cell line to different concentrations of 5-ALA and U0126 (Fig. 3). PpIX accumulation and 5-ALA-PDT-induced cell death increased in a concentration-dependent manner when DLD-1 cells were treated with increasing concentrations of 5-ALA (0.5‒5 mM), as expected (Fig. 3a). Treatment with MEK inhibitor, U0126 (2.5–200 µM), did not affect the cellular PpIX fluorescence in DLD-1 cells. Furthermore, no cell death was observed in cells that were treated solely with low concentrations of U0126 (0–20 µM), while increasing the concentration of U0126 beyond 25 µM induced significant death in DLD-1 cells (Fig. 3b). Next, we determined the efficacy of different sub-lethal concentrations of U0126 combined with 0.5 mM 5-ALA, a sub-effective concentration for 5-ALA-PDT, in DLD-1 cells (Fig. 3a, right). U0126-pre-treatment increased 5-ALA-induced PpIX accumulation in a concentration-dependent manner (Fig. 3c). Furthermore, while cell death was not observed with lower concentrations of U0126 (2.5 and 5 µM), combined treatment with higher concentrations of U0126 (10 and 20 µM) significantly promoted the efficacy of 5-ALA-PDT. Similar effects of U0126 pre-treatment were observed with a higher concentration of 5-ALA (5 mM). 5-ALA-PDT killed 98% of DLD-1 cells pre-treated with 20 µM U0126 (Fig. 3d).

To determine whether the increased efficacy of the combined treatment is through synergistic or additive drug interactions, we analysed our results shown in Fig. 3 using the Chou and Talalay method. The CI of all combinations of MEK inhibitor and 5-ALA concentrations tested was less than one (Supplementary Table 1), indicating that MEK inhibition synergistically enhanced the efficacy of 5-ALA-PDT.

### MEK inhibition promotes 5-ALA-PDT-induced ROS generation and PCD

**Fig. 4:**
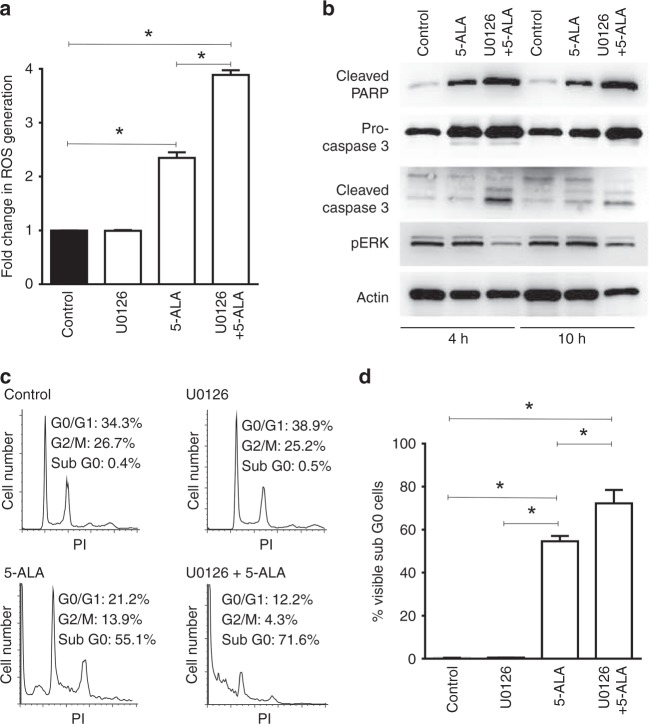
MEK inhibition enhances 5-ALA-PDT-induced ROS generation and programmed cell death (PCD). **a** Fold change in cellular ROS after PDT in DLD-1 cells relative to untreated control (black bar). **b** Representative western blot of apoptotic markers (cleaved PARP, pro-caspase 3 and cleaved caspase 3) conducted on cell lysates obtained at 4 h and 10 h after 5-ALA-PDT. P-ERK is a substrate of MEK which was used to confirm the effect of U0126, and actin was used as the loading control. **c** Representative cell cycle histograms of DLD-1 cells treated with U0126, 5-ALA and U0126 + 5-ALA followed by irradiation. PI propidium iodide. **d** Quantitative analysis of the sub-G0 populations in each treatment group. The data presented as mean ± SD from three independent experiments. *p < 0.01 by one-way ANOVA with Turkey’s post hoc test

5-ALA-PDT induces PCD in cancer cells through the generation of ROS.^5^ To determine whether MEK inhibition increases ROS generation after 5-ALA-PDT, we monitored the conversion of DCFH-DA to DCF in DLD-1 cells treated with control vehicle, U0126, 5-ALA-PDT or U0126/5-ALA-PDT (Fig. 4a). Cellular esterases deacetylate DCFH-DA to non-fluorescent-DCFH, which is rapidly oxidised to highly fluorescent DCF by ROS. Higher fluorescence was observed in 5-ALA-PDT-treated cells than in control or U0126-treated cells, indicating that 5-ALA-PDT-induced ROS generation. Moreover, combined 5-ALA-PDT with U0126 significantly increased ROS generation compared with that in cells treated with 5-ALA-PDT alone.

To determine whether MEK inhibition promotes PCD induction by 5-ALA-PDT, we first examined the activation of PCD markers by western blot analysis (Fig. 4b). An increase in cleaved PARP, pro-caspase 3 and caspase 3 was observed in cells treated with U0126+5-ALA-PDT compared with those in cells treated solely with 5-ALA-PDT at both 4 and 10 h after treatment, indicating that the activation of the cellular PCD pathways by 5-ALA-PDT was promoted by MEK inhibition. Reduced ERK phosphorylation (p-ERK) was observed in cells treated with U0126, demonstrating that U0126 inhibited the activation of the Ras/MEK pathway effectively.

We next assessed DNA fragmentation, a hallmark of PCD, by flow cytometry. Cells undergoing PCD have low-molecular-weight DNA fragments and cluster as the sub-G0 population. No significant G0 population was observed in the control group (0.4%) or the U0126-treated group (0.5%) (Fig. 4c). In contrast, the 5-ALA-PDT group showed a significant sub-G0 cell population (55%), which was further increased by combined 5-ALA-PDT with U0126 (70%) (Fig. 4c, d).

**Fig. 5:**
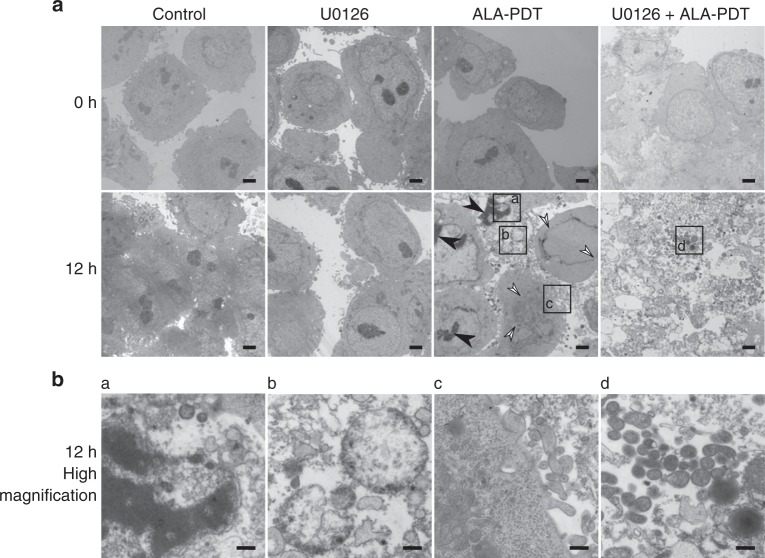
Combined MEK inhibition with 5-ALA-PDT promotes programmed cell death (PCD). **a** Representative low-magnification electron micrographs of DLD-1 cells at 0 and 12 h after PDT (scale bar = 2 µm). Nuclear condensation and nuclear membrane disruption are marked with black and white arrowheads, respectively. **b** Higher magnification micrographs of areas marked in **a** (scale bar = 500 nm). **a** nuclear condensation, **b** mitochondrial swelling, **c** cell membrane blebbing and **d** mitochondrial pyknosis

Lastly, the ultrastructure of the cells was examined by electron microscopy to find morphological changes typical of cells undergoing PCD (Fig. 5). Salient features of PCD, including nuclear condensation, mitochondrial swelling and cell membrane blebbing were not observed in either control DLD-1 cells or those treated with U0126. In contrast, these features were evident in DLD-1 cells 12 h after 5-ALA-PDT, and were more pronounced in cells treated with the combined treatment. Furthermore, cells subjected to the combined treatment showed extensive cell lysis with the disruption of the cell and nuclear membranes, mitochondrial pyknosis and the release of intracellular content, including membrane-bound organelles at 12 h post irradiation.

Taken together, these results suggest that MEK inhibition increases ROS generation, which in turn promotes PCD induction in cells treated with 5-ALA-PDT. This increase in PCD was evident from drastic changes in cell morphology (Fig. 5), and the increased sub-G0 population (Fig. 4c, d) in the combined treatment group.

### MEK inhibition promoted 5-ALA-PDT efficacy in vivo

**Fig. 6:**
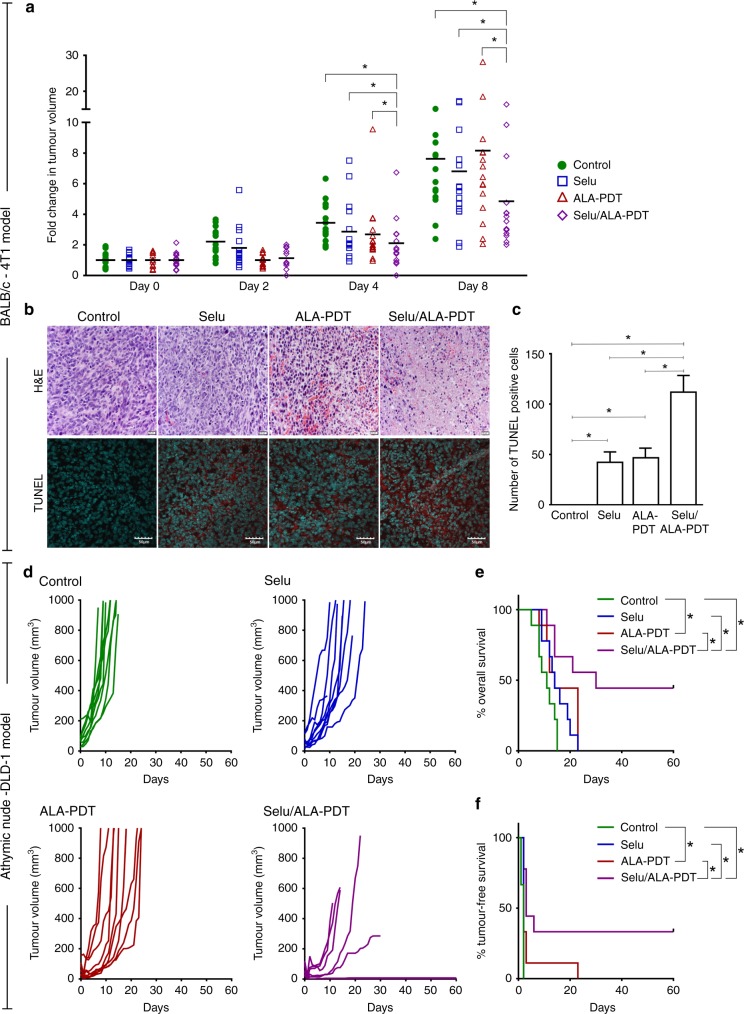
MEK inhibition promotes 5-ALA-PDT efficacy in mouse cancer models. **a**–**c** BALB/c mice-bearing mouse mammary 4T1 tumours; **d**–**f** athymic nude mice-bearing human colon cancer DLD-1 tumours. **a** On day 0, BALB/c mice-bearing 4T1 breast tumour (*n* = 15/group) were treated with selumetinib (250 mg/kg p.o.), 5-ALA (200 mg/kg i.p.) and PDT. Fold changes in tumour volume relative to the mean volume on day 0 in each treatment group are shown. Horizontal bars represent the mean of each group. *p < 0.05 by two-way ANOVA with Turkey’s post hoc test. **b** Representative histological images of tumours from BALB/c mice at 48 h post treatment. The top panel shows H&E staining, and the bottom panel shows TUNEL and DAPI staining (red: TUNEL-positive, blue: nuclei). **c** Quantitative analysis of TUNEL-positive cells in each treatment group (three mice/group). *p < 0.05 by one-way ANOVA with Turkey’s post hoc test. **d** Athymic nude mice (*n* = 9/group) bearing DLD-1 human colon tumours were treated with selumetinib, 5-ALA and PDT. Tumour volumes of individual athymic nude mice are shown. Note that in Selu/ALA-PDT, three mice did not have any tumour, and another one had a very small tumour. Their plots can be seen very close to the *x*-axis. **e** Kaplan–Meier overall survival curves and **f** Kaplan–Meier tumour-free survival curves for the four groups. *p < 0.05 by log-rank test

To determine whether these in vitro findings were also true in vivo, we examined the effectiveness of the combined 5-ALA-PDT with a MEK inhibitor in animal models of cancer. For in vivo experiments, we chose selumetinib instead of U0126 as this MEK inhibitor is currently under phase II clinical trial for various cancers and is more suitable for animal studies.^28,38^ We first tested the 5-ALA-PDT sensitivity of mouse 4T1 mammary carcinoma cell line in vitro and found that MEK inhibition increased 5-ALA-PDT efficacy in 4T1 murine cells similar to the effect seen in human cancer cell lines (Supplementary Fig. S2). We also confirmed the in vitro efficacy of combined treatment with selumetinib and 5-ALA-PDT in 4T1 and DLD-1 cells (Supplementary Fig. S2). Next, BALB/c mice-bearing 4T1 tumours were randomly divided into four groups—vehicle control, selumetinib, 5-ALA-PDT and selumetinib/5-ALA-PDT. Selumetinib or 5-ALA-PDT monotherapy did not affect tumour growth (Fig. 6a). However, tumour growth in the combined selumetinib/5-ALA-PDT group was significantly slower than those in the vehicle control, selumetinib, or 5-ALA-PDT groups up to 8 days post treatment (Fig. 6a). We did not observe any significant differences after 8 days post treatment as 4T1 mammary tumours grow aggressively and have a high-proliferation rate obscuring the antitumor effects of selumetinib/5-ALA-PDT. H&E staining of tumour tissues revealed higher numbers of dead and non-nucleated cells in the selumetinib/5-ALA-PDT group compared with those in other groups (Fig. 6b). TUNEL staining, which detects DNA fragmentation, revealed a significant increase in TUNEL-positive cells, indicating that PCD was more actively induced in the selumetinib/5-ALA-PDT group, compared with either of the monotherapies (Fig. 6b, c).

We also determined the efficacy of combined 5-ALA-PDT with selumetinib on human colon DLD-1 tumours in a mouse xenograft model. Mice were divided into four experimental groups, as previously indicated (Fig. 6d). All mice in the vehicle control group attained the maximum tumour size and were killed by day 17. Although the growth of tumours in mice treated with selumetinib or 5-ALA-PDT monotherapy was slower compared with those treated with the control vehicle, no mice survived after day 23. In contrast, three of the nine mice that received combined 5-ALA-PDT with selumetinib showed complete remission, and no tumour was observed at 60 days after treatment. In addition, another mouse carried a very small tumour (<10 mm^3^), which did not show any further growth. The overall survival rate of the selumetinib/5-ALA-PDT group was 44% (four out of nine mice) (Fig. 6e), and the tumour-free survival was 33% (three out of nine mice) (Fig. 6f). Kaplan–Meier survival analyses demonstrated that combined 5-ALA-PDT with selumetinib significantly promoted the overall and tumour-free survival compared with treatment with selumetinib or 5-ALA-PDT alone.

These results from animal models of mouse mammary and human colon cancers demonstrate that systemic inhibition of the Ras/MEK pathway significantly improves the in vivo therapeutic efficacy of 5-ALA-PDT.

## Discussion

While 5-ALA-PDT is an attractive therapeutic option for localised tumours due to its specificity and limited side effects, low PpIX accumulation in the tumour results in a high rate of incomplete treatment response and disease relapse.^12–15,39,40^ Thus, a tumour-specific increase in PpIX accumulation should improve the efficacy and utility of 5-ALA-PDT. Based on our previous study demonstrating that MEK inhibition increases PpIX accumulation in cancer cells,^27^ this study sought to determine whether MEK inhibition would increase 5-ALA-PDT efficacy in vitro and in vivo. We found that MEK inhibition increased the sensitivity of various cancer cell lines to 5-ALA-PDT in vitro. Furthermore, we demonstrated that 5-ALA-PDT combined with a MEK inhibitor was more effective than 5-ALA-PDT monotherapy in animal models of breast and colon cancer.

Most importantly, a complete response was achieved in 44% of the human colon cancer xenograft model mice that received the combined 5-ALA-PDT and MEK inhibitor. These results are directly relevant for improving the efficacy of 5-ALA-PDT in clinical settings. MEK inhibitors such as trametinib and cobimetinib are approved for clinical use for different types of cancers,^28^ and are considered relatively safe with manageable side effects such as rashes, diarrhoea, peripheral oedema, fatigue and mild retinopathy.^41,42^ Therefore, it would be feasible to combine a MEK inhibitor with 5-ALA-PDT to reduce incomplete treatment responses and disease relapse in patients.

One of the critical findings of this study is that Ras/MEK activation regulates the 5-ALA-PDT sensitivity of tumours. Activating mutations of Ras protein are found in ~30% of all human tumours.^21^ Even in cancers where an activating mutation of Ras protein is absent, the upstream or downstream signalling components of the Ras/MEK pathway are often inappropriately activated. Owing to this, >80% of cancer cells are considered to have a constitutively activated Ras/MEK pathway.^20,22–24^ This suggests that the Ras/MEK pathway plays a critical role in defining the cancer cell sensitivity to 5-ALA-PDT in a broad range of tumours regardless of their type or origin. The Ras/MEK pathway has multiple downstream elements that regulate signalling modifications and translational and transcriptional activities. It remains to be identified which downstream elements are involved in the regulation of PpIX accumulation. Once identified, these downstream elements may be used as novel biomarkers for accurately predicting 5-ALA-PDT sensitivity in clinical settings. Furthermore, targeting these downstream elements instead of Ras/MEK may be a better therapeutic strategy, as the effect would probably be more specific and have fewer off-target effects.

Although the increase in PpIX accumulation is likely the main factor contributing to the enhancement of 5-ALA-PDT efficacy by MEK inhibition, other effects of MEK inhibitors may also be involved. The Ras/MEK pathway plays critical roles in cancer cell death.^21,23,24^ Accordingly, the activated Ras pathway protects cells from ROS-induced cellular oxidation by increasing the expression of several antioxidant proteins.^43^ Interestingly, 5-ALA-PDT also activates the Ras/MEK pathway, which in turn reduces the induction of PCD in squamous carcinoma cells treated with 5-ALA-PDT.^44^ Therefore, MEK inhibition may reduce the expression of antioxidant proteins and promote cancer cell death induced by 5-ALA-PDT. Moreover, as oncogenic activation of Ras/MEK promotes cellular proliferation via transcriptional and translational regulation,^20^ MEK inhibition may also directly suppress in vivo tumour growth. Finally, treatment with a MEK inhibitor promotes antitumor immunity by modulating the expression of programmed death-ligand 1 (PD-L1),^45^ which might contribute to the increased efficacy of 5-ALA-PDT combined with the MEK inhibitor in an immunocompetent tumour model.

In moderately and least sensitive cell lines, we found that MEK inhibition increased the 5-ALA-PDT sensitivity to a certain extent; however, a complete sensitisation to the level of the sensitive cell lines was not achieved. This suggests that other cellular mechanisms independent from oncogenic Ras/MEK may be involved in regulating 5-ALA-PDT sensitivity of cancer cells. Various membrane transporters such as ABCB1, ABCB6 and ABCG2 have been shown to regulate cellular PpIX levels.^46,47^ In our previous study, we found that Ras/MEK regulates the expression of ABCB1, but not ABCB6 or ABCG2.^27^ Therefore, it is likely that other membrane transporters contribute to 5-ALA-PDT sensitivity independently of Ras/MEK. The subcellular localisation of the PS also contributes to the cellular sensitivity to PDT, as PDT-resistant cells have been shown to accumulate low amounts of PS in the mitochondria.^48^ Furthermore, some cancer cells develop resistance against PCD initiated by PDT-induced cellular oxidation.^49,50^ How these factors add to or interact with the Ras/MEK-dependent mechanisms and affect the cellular sensitivity to 5-ALA-PDT requires further investigation.

Since 5-ALA-PDT as a monotherapy often fails to achieve satisfactory clinical outcomes for treating cancer patients, combination therapies with distinct modes of action may provide better treatment efficacy and disease management. Several combination strategies have been tested thus far to enhance 5-ALA-PDT efficacy, such as treatment with ABC transporter inhibitors, FECH inhibitors, chemotherapy agents and vitamin D.^40,46,51–54^ In this study, we demonstrate an improved efficacy of 5-ALA-PDT combined with MEK inhibitors using preclinical models. As MEK inhibitors increase the PpIX accumulation in tumours by downregulation of both ABCB1 and FECH,^27^ they may be superior to inhibitors of ABC transporters or FECH in enhancing 5-ALA-PDT. Furthermore, MEK inhibitors can be expected to have additional inhibitory effects on cancer. Therefore, our results warrant a clinical trial for the combined MEK inhibitor/5-ALA-PDT as a safe and effective strategy for improving cancer treatment.

As there are several MEK inhibitors approved for human use, there would be minimal regulatory hurdles for a clinical trial. Since PDT is a treatment modality that involves several components, including the PS, PS dose, light source, light energy, drug–light–interval and irradiation protocols, it is critical to optimise all variables and establish a treatment plan for obtaining maximal disease clearance.^9^ Furthermore, the interaction between the MEK inhibitor and pre-existing conditions and/or medications of patients should be considered. It is also essential to establish clear advantages of this novel treatment over conventional strategies in large controlled comparative randomised clinical trials. With the establishment of optimal protocols, MEK inhibition combined with 5-ALA-PDT can prove to be an ideal treatment strategy that is minimally invasive and effective for various cancers.

## Supplementary information

Supplementary figures and table

## Data Availability

All data generated or analysed during this study are included in this published article.
