# Supplementary material for: Systemic MEK inhibition enhances the efficacy of 5-aminolevulinic acid-photodynamic therapy
Source: Br J Cancer. 2019 Sep 25;121(9):758–67. doi: 10.1038/s41416-019-0586-3 (PMC6889170; doi:10.1038/s41416-019-0586-3)
Supplement: Supplementary file 1 — Supplementary figures and table [file 41416_2019_586_MOESM1_ESM.pdf]

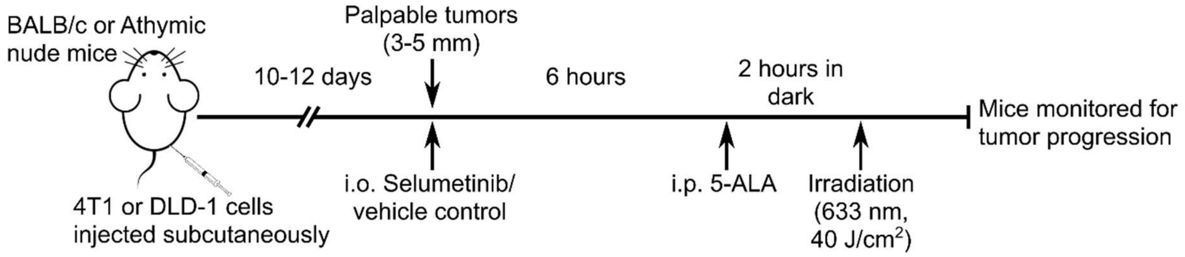

Figure S1. Schematic representation of animal treatment.

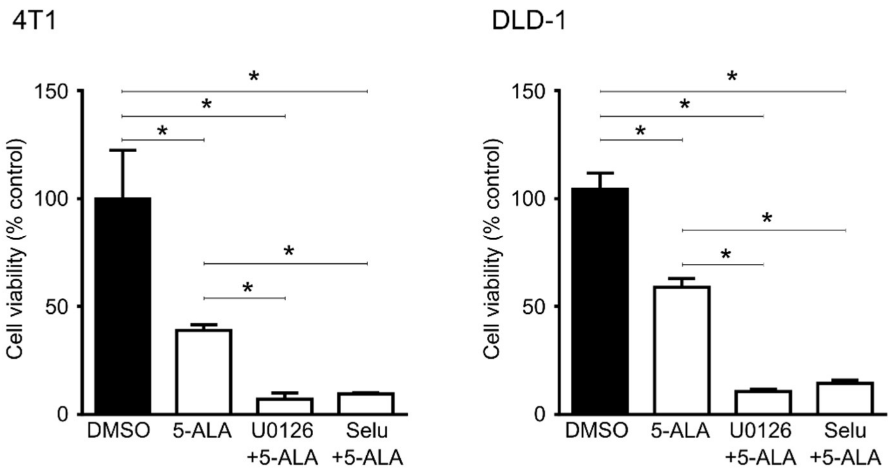

Figure S2. **MEK inhibitors promote cell death by 5-ALA-PDT in cancer cell lines *in vitro*.** 4T1 and DLD-1 cells were treated with or without MEK inhibitors U0126 (20 μM) or selumetinib (20 μM) for 20 h and then with 5-ALA (5 mM) for 4 h followed by irradiation. Mean ± SD of % Cell viability at 24 h after 5-ALA-PDT compared to control (DMSO, black bar) from three independent experiments is shown. *p<0.01 by one-way ANOVA with Turkey's posthoc test.

5-ALA (mM)	U0126 (μM)	CI
0.5	2.5	**0.60**
0.5	5.0	**0.49**
0.5	10.0	**0.28**
0.5	20.0	**0.12**
5.0	2.5	**0.90**
5.0	5.0	**0.83**
5.0	10.0	**0.62**
5.0	20.0	**0.06**

Supplementary Table 1. Chou and Talalay analysis of the interaction between 5-ALA PDT and MEK inhibitor U0126. The table shows the combination indices (CI) for the treatment combinations shown in Figure 3.
